# Supplementary material for: Genetic and environmental contributions to epigenetic aging across adolescence and young adulthood
Source: Clin Epigenetics. 2025 May 7;17:78. doi: 10.1186/s13148-025-01880-6 (PMC12060359; doi:10.1186/s13148-025-01880-6)
Supplement: Supplementary file 1 — Supplementary material 1: The SNP-, pedigree-, and twin-based heritability of epigenetic aging estimated in previous studies. [file 13148_2025_1880_MOESM1_ESM.docx]

**Additional file 1: Previously published estimates of heritability of the analyzed epigenetic aging measures**

**Table S1**

*Estimates of heritability of epigenetic aging measures from twin-, SNP-, and pedigree-based studies*

| Study | *M* (*SD*/Range) | Heritability | Twin pairs | Individuals | Measure | Method |
| --- | --- | --- | --- | --- | --- | --- |
| Horvath, 2013 [2] | 0 (0-0) | 1.00 | - | 53 | Horvath Accel. | Twin |
| Horvath, 2013 [2] | 63^1^ (45-75) | 0.39 | - | 93 | Horvath Accel. | Twin |
| Levine et al., 2015 [6] | 89 (6.4) | 0.41^5^ | - | 700 | Horvath Accel. | SNP |
| Marioni et al., 2015 [8] | 14^2^ (9-23)  47^3^ (33-75) | 0.43 | 178 | 614 | Horvath Diff. | Pedigree (twins, siblings, parents) |
| Lu et al., 2016 [7] | 48 (16-96) | 0.69/ 0.01/0.01/0.59^6^ | - | 112 | Horvath Accel. | SNP |
| Lu et al., 2016 [7] | 52 (1-102) | 0.15/0.14^7^ | - | 201 | Horvath Accel. | SNP |
| Simpkin et al., 2016 [11] | 15^4^ (15-NA) | 0.37 | - | 2 036 | Horvath Accel. | SNP (mothers, children) |
| Jylhävä et al., 2019 [3] | 70 (8.4) | 0.55 | 104 | 208 | Horvath Accel. | Twin |
| Jylhävä et al., 2019 [3] | 79 (8.3) | 0.51 | 104 | 208 | Horvath Accel. | Twin |
| Sillanpää et al., 2019 [10] | 23 (0.9) | 0.74 | 289 | 578 | Horvath Accel. | Twin |
| Sillanpää et al., 2019 [10] | 61 (3.7) | 0.53 | 164 | 328 | Horvath Accel. | Twin |
| Kankaanpää et al., 2021 [4] | 22 (0.7) | 0.69 | 285 | 570 | Horvath Accel. | Twin |
| Kankaanpää et al., 2021 [4] | 62 (4.1) | 0.61 | 235 | 470 | Horvath Accel. | Twin |
| Kankaanpää et al., 2021 [4] | 22 (0.7) | 0.62 | 285 | 570 | GrimAge Accel. | Twin |
| Kankaanpää et al., 2021 [4] | 62 (4.1) | 0.58 | 235 | 470 | GrimAge Accel. | Twin |
| Jylhävä et al., 2019 [3] | 70 (8.4) | 0.55 | 104 | 208 | Horvath Accel. | Twin |
| Jylhävä et al., 2019 [3] | 79 (8.3) | 0.51 | 104 | 208 | Horvath Accel. | Twin |
| Kankaanpää et al., 2022 [5] | 22 (0.7) | 0.73 | 365 | 730 | GrimAge Accel. | Twin |
| Kankaanpää et al., 2022 [5] | 22 (0.7) | 0.62 | 365 | 730 | DunedinPoAm | Twin |
| Kankaanpää et al., 2022 [5] | 22 (0.7) | 0.68 | 365 | 730 | DunedinPACE | Twin |
| Hong et al., 2024^8^ [1] | 31 (31-31) | 0.66 |  | 986 | DunedinPACE | Twin |
| Hong et al., 2024 [1] | 70 (70-70) | 0.44 |  | 986 | DunedinPACE | Twin |
| Hong et al., 2024 [1] | 31 (31-31) | 0.76 |  | 986 | PC Horvath Accel. | Twin |
| Hong et al., 2024 [1] | 70 (70-70) | 0.49 |  | 986 | PC Horvath Accel. | Twin |
| Hong et al., 2024 [1] | 31 (31-31) | 0.60 |  | 986 | PC GrimAge Accel. | Twin |
| Hong et al., 2024 [1] | 70 (70-70) | 0.55 |  | 986 | PC GrimAge Accel. | Twin |
| Miao et al., 2024 [9] | 50.24 (26-77) | 0.69 | 134 | **268** | DunedinPACE | Twin |
| Miao et al., 2024 [9] | 54.87 (31-82) | 0.72 | 134 | **268** | DunedinPACE | Twin |
| Miao et al., 2024 [9] | 50.24 (26-77) | 0.70 | 134 | **268** | GrimAge Accel. | Twin |
| Miao et al., 2024 [9] | 54.87 (31-82) | 0.69 | 134 | **268** | GrimAge Accel. | Twin |

*Note.* SNP, single nucleotide polymorphisms-based heritability analysis; Pedigree, pedigree-based heritability analysis; Twin, patterns of genetic resemblance between monozygotic and dizygotic twins; *M*, mean age; *SD*, standard deviation; NA, not available; Accel., the residuals from a linear regression model of epigenetic age on chronological age; Diff., the difference between epigenetic age and chronological age, PC, principal components from the CpG sites derived from the correspondent epigenetic aging measure.

^1^ Median of age.

^2^ Mean age of children.

^3^ Mean age of parents.

^4^ Oldest age combination (15-year-old children and middle age mothers).

^5^ Estimates of heritability for cerebellum/frontal cortex/pons/temporal cortex.

^6^ Estimates of heritability for cerebellum/frontal cortex.

^7^ Estimates of heritability for dorsolateral prefrontal cortex.

^8^ Estimates for the youngest and for the oldest age groups. The heritability estimates for epigenetic aging measures were derived in local structural equation modeling. The total sample size for ages between 30 and 70 is 986, with a raw sample size of 16 at age 30 and 8 at age 70.

**References:**

1. Hong X, Cao H, Cao W, Lv J, Yu C, Huang T, et al. Trends of genetic contributions on epigenetic clocks and related methylation sites with aging: A population‐based adult twin study. Aging Cell. 2024;e14403. <https://doi.org/10.1111/acel.14403>
2. Horvath S. DNA methylation age of human tissues and cell types. Genome Biology. 2013;14(10):R115. <https://doi.org/10.1186/gb-2013-14-10-r115>
3. Jylhävä J, Hjelmborg J, Soerensen M, Munoz E, Tan Q, Kuja-Halkola R, et al. Longitudinal changes in the genetic and environmental influences on the epigenetic clocks across old age: Evidence from two twin cohorts. EBioMedicine. 2019;40:710–6. <https://doi.org/10.1016/j.ebiom.2019.01.040>
4. Kankaanpää A, Tolvanen A, Bollepalli S, Leskinen T, Kujala UM, Kaprio J, et al. Leisure-Time and Occupational Physical Activity Associates Differently with Epigenetic Aging. Medicine & Science in Sports & Exercise. 2021;53(3):487–95. <https://doi.org/10.1249/MSS.0000000000002498>
5. Kankaanpää A, Tolvanen A, Heikkinen A, Kaprio J, Ollikainen M, Sillanpää E. The role of adolescent lifestyle habits in biological aging: A prospective twin study. eLife. 2022;11:e80729. <https://doi.org/10.7554/eLife.80729>
6. Levine ME, Lu AT, Bennett DA, Horvath S. Epigenetic age of the pre-frontal cortex is associated with neuritic plaques, amyloid load, and Alzheimer’s disease related cognitive functioning. Aging. 2015;7(12):1198–1211. <https://doi.org/118632/aging.100864>
7. Lu AT, Hannon E, Levine ME, Hao K, Crimmins EM, Lunnon K, et al. Genetic variants near MLST8 and DHX57 affect the epigenetic age of the cerebellum. Nature Communications. 2016;7(1):10561. <https://doi.org/11038/ncomms10561>
8. Marioni RE, Shah S, McRae AF, Chen BH, Colicino E, Harris SE, et al. DNA methylation age of blood predicts all-cause mortality in later life. Genome Biology. 2015;16(1):25. https://doi.org/10.1186/s13059-015-0584-6
9. Miao K, Liu S, Cao W, Lv J, Yu C, Huang T, et al. Five years of change in adult twins: longitudinal changes of genetic and environmental influence on epigenetic clocks. BMC Med. 2024;22(1):289. https://doi.org/10.1186/s12916-024-03511-y
10. Sillanpää E, Ollikainen M, Kaprio J, Wang X, Leskinen T, Kujala UM, et al. Leisure-time physical activity and DNA methylation age—A twin study. Clinical Epigenetics. 2019;11(1):12. <https://doi.org/10.1186/s13148-019-0613-5>
11. Simpkin AJ, Hemani G, Suderman M, Gaunt TR, Lyttleton O, Mcardle WL, et al. Prenatal and early life influences on epigenetic age in children: A study of mother–offspring pairs from two cohort studies. Human Molecular Genetics. 2016;25(1):191–201. https://doi.org/10.1093/hmg/ddv456
